# Identification of a Novel HIV-1 Circulating Recombinant Form (CRF72_BF1) in Deep Sequencing Data from Blood Donors in Southeastern Brazil

**DOI:** 10.1128/genomeA.00386-14

**Published:** 2014-06-12

**Authors:** Rodrigo Pessôa, Anna Bárbara de Freitas Carneiro Proietti, Michael P. Busch, Sabri S. Sanabani

**Affiliations:** a Department of Virology, São Paulo Institute of Tropical Medicine, University of São Paulo, São Paulo, Brazil; b Foundation and Center for Hematology and Hemotherapy of Minas Gerais, Hemominas, Belo Horizonte, MG, Brazil; c Blood Systems Research Institute, San Francisco, California, USA; d Department of Pathology, LIM 03, Hospital das Clínicas, School of Medicine, University of São Paulo, São Paulo, Brazil

## Abstract

We report the identification of a novel HIV-1 circulating recombinant form (CRF72_BF1) in deep sequencing data from peripheral blood mononuclear cells (PBMC) of five blood donors in southeastern Brazil. Detection of this circulating recombinant form (CRF) confirms the need for effective surveillance to monitor the prevalence and distribution of HIV variants in a variety of settings in Brazil.

## GENOME ANNOUNCEMENT

Both high mutation rates and recombination significantly contribute to the genetic diversification of human immunodeficiency virus type 1 (HIV-1). To date, HIV-1 viruses are classified into four phylogenetic groups, M, O, N, and P. The M group is further subdivided into nine subtypes (A to D, F to H, J, and K), among which subtypes A and F have been further classified into sub-subtypes (1, 2). Recombinant strains from at least three unlinked epidemiological sources, which exhibit identical mosaic patterns, have been classified separately as circulating recombinant forms (CRFs) (3). Recent estimates show that the HIV-1 CRFs and other minor recombinants account for approximately 20% of global HIV-1 infections (4). Here, we report the near full-length genome sequences (NFLGs) of a novel HIV-1 BF1 recombinant, designated CRF72_BF1 by the Los Alamos National Laboratory, derived from five blood donors in Minas Gerais, southeastern Brazil.

Cellular DNA was extracted from 5 peripheral blood mononuclear cells (PBMC) using the QIAamp blood kit (Qiagen) according to the manufacturer's instructions. The NFLGs from five overlapping fragments were obtained by PCR and determined by a previously reported method (5). The sequencing library was prepared as described previously (6). Briefly, the amplified fragments from a single viral genome were purified, quantified, and pooled at equimolar ratios. Approximately 1 ng of each pool was used in a fragmentation reaction. Finally, all libraries were pooled and loaded on an Illumina MiSeq for paired-end 250 sequencing. Fastq files were generated, validated, and *de novo* assembled into contiguous sequences and annotated with CLC Genomics Workbench version 5.5. The assembled contiguous sequences were aligned with reference sequences and screened for recombination by the bootscan methods (7, 8). Maximum likelihood trees were obtained by PhyML v. 3.1 using the GTR + I + G model (9). The approximate likelihood ratio test was used as a statistical test to calculate branch support.

The ultradeep sequencing yielded over 1.6 × 10^6^ sequences reads, with average coverage ranging from 567× (10BR_MG025) to 14,333× (10BR_MG004). The NFLG consensus sequence from each strain of five BF1 (10BR_MG002, 10BR_MG003, 10BR_MG004, 10BR_MG008, and 10BR_MG025) was initially investigated using the bootscan method, which showed them to display identical mosaic structures, with 10 intersubtype breakpoints between subtype B and subclade F1 in *gag* (1 breakpoint), *pol* (4 breakpoints), *vif* (1 breakpoint), *env* (2 breakpoints), *rev2* (1 breakpoint), and *nef* (1 breakpoint), and with 6 subtype B regions and 5 subclade F1 regions. Comparison with published sequences revealed an additional two HIV sequences from Southeast Brazil (99UFRJ-1 and BREPM1029) that share an identical mosaic structure with those reported in our study (10, 11). Reconstructed trees for each region corroborated the results from the bootscan. The genetic distances between the CRF72_BF1 sequences exceed 8%, suggesting their longstanding circulation in Southeast Brazil.

The availability of the new CRF72_BF1 sequences described in this study should contribute to a more robust understanding of the overall genetic variability and phylogenetic relationships within and among other group M subtypes. Further molecular epidemiological investigations in a variety of settings are needed to identify the influence of CRF72_BF1 on the HIV epidemic in Brazil.

### Nucleotide sequence accession numbers.

All consensus genome assemblies generated in this study were submitted to NCBI’s GenBank database under accession numbers KJ671533 to KJ671537.
